# Impact of Tylosin Tartrate and Ciprofloxacin on the Deposition of Negatively Charged Polystyrene Nanoparticles onto SiO_2_

**DOI:** 10.1021/acs.langmuir.5c01405

**Published:** 2025-07-15

**Authors:** Anna L. DiFelice, Anna Silver, Elizabeth A. Good, Arielle C. Mensch

**Affiliations:** † Department of Chemistry, 3091 Lafayette College, Easton, Pennsylvania 18042, United States

## Abstract

Aqueous micropollutants can enter the environment through the degradation of macropollution and through ineffective wastewater treatment. Nanoparticles (NPs) and antibiotics, two classes of micropollutants, are of particular concern because of their increased reactivity and potential toxicological effects. The high surface area-to-volume ratio of NPs makes them susceptible to the sorption of other organic contaminants, including antibiotics. The environmental transformations that NPs undergo complicate the determination of where they and any adsorbed organic contaminants may accumulate in the environment. This work aims to investigate the role of surface termination in the environmental transformations and deposition of polystyrene nanoparticles (PSNPs) in varying ionic strength and antibiotic matrices. We used two negatively charged PSNP model systems, carboxyl (COOH-PSNPs)- and sulfate (SO_4_-PSNPs)-terminated PSNPs, and two different antibiotic model systems, tylosin (TYL) tartrate and ciprofloxacin (CIP). Increasing the concentration ratio of TYL to PSNPs impacted the hydrodynamic and electrokinetic properties of the PSNPs in surface termination-, ionic strength-, and concentration-dependent manners. High concentrations of TYL induced aggregation of the SO_4_-PSNPs and resulted in an increased ζ potential at high ionic strengths for both particle types, whereas CIP had minimal impacts on both types of particles across ionic strength conditions. Using a quartz crystal microbalance to investigate the deposition of both pristine and transformed PSNPs onto a model sediment surface, SiO_2_, we observed more deposition of SO_4_-PSNPs than of COOH-PSNPs at high ionic strengths. The presence of TYL eliminated attachment of the SO_4_-PSNPs and had a negligible impact on the deposition of COOH-PSNPs, whereas CIP increased attachment of the SO_4_-PSNPs and had a negligible impact on the deposition of the COOH-PSNPs. Together, these results highlight the importance of surface termination and micropollutant physicochemical properties in understanding environmental transformations that NPs may undergo and the subsequent environmental consequences they may have.

## Graphical abstract

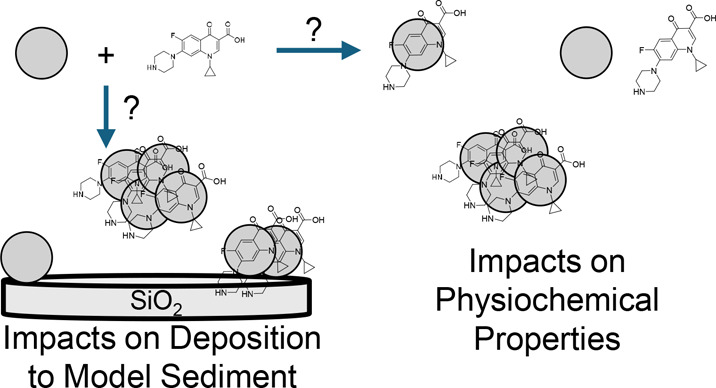

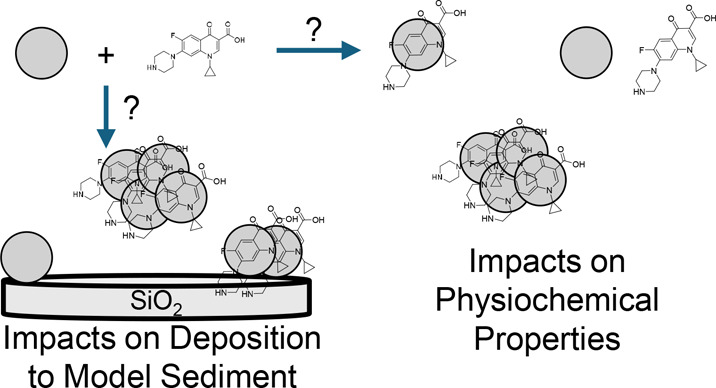

## Introduction

Chemical pollution of freshwater environments is a global problem that has largely unknown consequences for aquatic life and human health. Aqueous micropollutants are defined as substances found in low concentrations that contribute to global water pollution concerns and can include pharmaceuticals, personal care products, heavy metals, industrial compounds, pesticides, plasticizers, nanoparticles, and microplastics.^1,2^ Concentrations of micropollutants range from nanograms to micrograms per liter,^1,2^ but despite their low concentrations, micropollutants are of particular concern due to their uncertain toxicological effects, their difficulties in being detected, and the lack of remediation technologies to remove them.^1^

These micropollutants do not exist in isolation, and their presence in natural environments may impact the behavior of other micropollutants.^3,4^ In particular, the high surface area-to-volume ratio of nanoparticles (NPs), which makes them more reactive than their bulk material counterparts, makes them particularly susceptible to undergoing environmental transformations^4–6^ in the presence of copollutants. A Trojan Horse model in which NPs may serve as a vector to transport other harmful organic pollutants via sorption and desorption throughout the environment has been suggested.^7–9^ In particular, the sorption processes dictating interactions between NPs and organic pollutants are hypothesized to be dependent on the physicochemical properties of both the NPs (e.g., surface chemistry, size, and surface charge) and the pollutants (e.g., size, hydrophobicity, and hydrogen bonding ability), as well as solution conditions such as pH and ionic strength.^10^ The sorption of organic pollutants to NPs may change the physicochemical properties of NPs, which ultimately can change where NPs end up within aqueous environments, and their aquatic toxicities.^11–13^

Previous studies have investigated the deposition of single pollutants onto model sediment surfaces, including investigating the deposition of antibiotics onto sediment surfaces such as goethite,^14^ montmorillonite and vermiculite,^15^ silica,^16,17^ and diatomaceous earth^18–20^ and that of NPs onto silica sand^21^ and silica and alumina surfaces.^22^ Some studies have looked at the deposition processes of NPs in the presence of other species such as natural organic matter,^23–25^ but less is known about NP deposition in the presence of antibiotic copollutants or the deposition of transformed pollutants onto model sediment surfaces.

The objectives of this work were to investigate the impact of an increasing antibiotic-to-NP concentration ratio on the environmental behavior of polystyrene NPs with different surface chemistries in varying ionic strength water chemistries. To achieve these objectives, we selected model micropollutants that could coexist in freshwater settings. Specifically, we chose polystyrene nanoparticles (PSNPs) with two different surface terminations (carboxyl (COOH-PSNPs) and sulfate (SO_4_-PSNPs)) as our model NP systems and the antibiotics tylosin (TYL) tartrate and ciprofloxacin (CIP) as our model antibiotic systems. PSNPs have been shown previously to sorb other organic and inorganic contaminants, including personal care products,^26–28^ antibiotics,^26,29–31^ polycyclic aromatic hydrocarbons,^3,32^ and metals.^33,34^ However, the subsequent environmental impacts of transformed PSNPs are not well understood, making them an important choice to study further. In addition, exploring both -COOH and -SO_4_ terminal groups provides model PSNP systems that are negatively charged and vary in size and charge density, allowing us to probe the role of surface chemistry rather than charge alone in driving interactions with other pollutants. The choice of TYL and CIP as antibiotic model systems was motivated by the environmental relevance of the two antibiotics,^35^ as well as their differing chemical properties. TYL is a common veterinary macrolide, whereas CIP is a common quinolone antibiotic used to treat human bacterial infections. With a p*K*_a_ value of 7.1,^36^ TYL will have positively charged amine groups at an environmentally relevant pH of 7.4. Conversely, CIP exhibits zwitterionic properties at a pH of 7.4, with the secondary cyclic amine protonated and the carboxylic acid deprotonated. Both of these model antibiotics allow a platform to probe the role of electrostatics in subsequent interactions with our negatively charged PSNPs. We used dynamic light scattering and laser Doppler microelectrophoresis to characterize the impact of TYL and CIP on the hydrodynamic and electrokinetic properties of PSNPs. In addition, we used a quartz crystal microbalance with dissipation monitoring (QCM-D) to investigate the interaction of NPs with a model sediment surface, SiO_2_, in the presence and absence of antibiotics. Understanding the deposition processes of PSNPs in the presence and absence of organic micropollutants can help us to better understand the complex behavior of PSNPs in more realistic water chemistries (e.g., in the presence of copollutants and at varying ionic strengths). The results presented here provide new insights into how the presence of copollutants alters the physicochemical behavior of NPs in a surface termination-dependent manner and highlights the need for additional studies aimed at elucidating the biological and environmental impacts of copollutants.

## Experimental Section

### Material Information for PSNPs and Their Purification by Dialysis

Sulfate-terminated polystyrene nanoplastics (SO_4_-PSNPs) were obtained from Sigma-Aldrich (LB-1). According to the manufacturer, the SO_4_-PSNPs are polystyrene latex beads with a 0.1 μm mean particle diameter and terminal sulfate groups are located on the particle surface.^37^ Carboxylated polystyrene latex NPs (COOH-PSNPs) were obtained from Magsphere, Inc. (CA100NM). The manufacturer reported a particle diameter of 97 ± 15 nm. Structures of the two types of PSNPs are shown in Figure 1. Both particle types were dialyzed prior to use, following previously published^38^ methods, to remove any sodium azide preservative and/or surfactants found in the suspensions that could interfere with subsequent studies. Briefly, the stock solution of PSNPs was dialyzed (Spectra7 dialysis membrane, MWCO 1000) against DI water for 5 days. The water was changed every 3 h for the first 12 h and every 12 h thereafter. Particles were parafilmed and stored at 4 °C in the dark when not in use. To confirm the sizes reported by the manufacturer, dilute solutions of NPs in isopropyl alcohol were drop-casted onto scanning electron microscopy (SEM) stubs, sputter-coated with gold, and imaged by using a Zeiss EVO SEM instrument with a SE1 detector (Figure S1).

**Figure 1:**
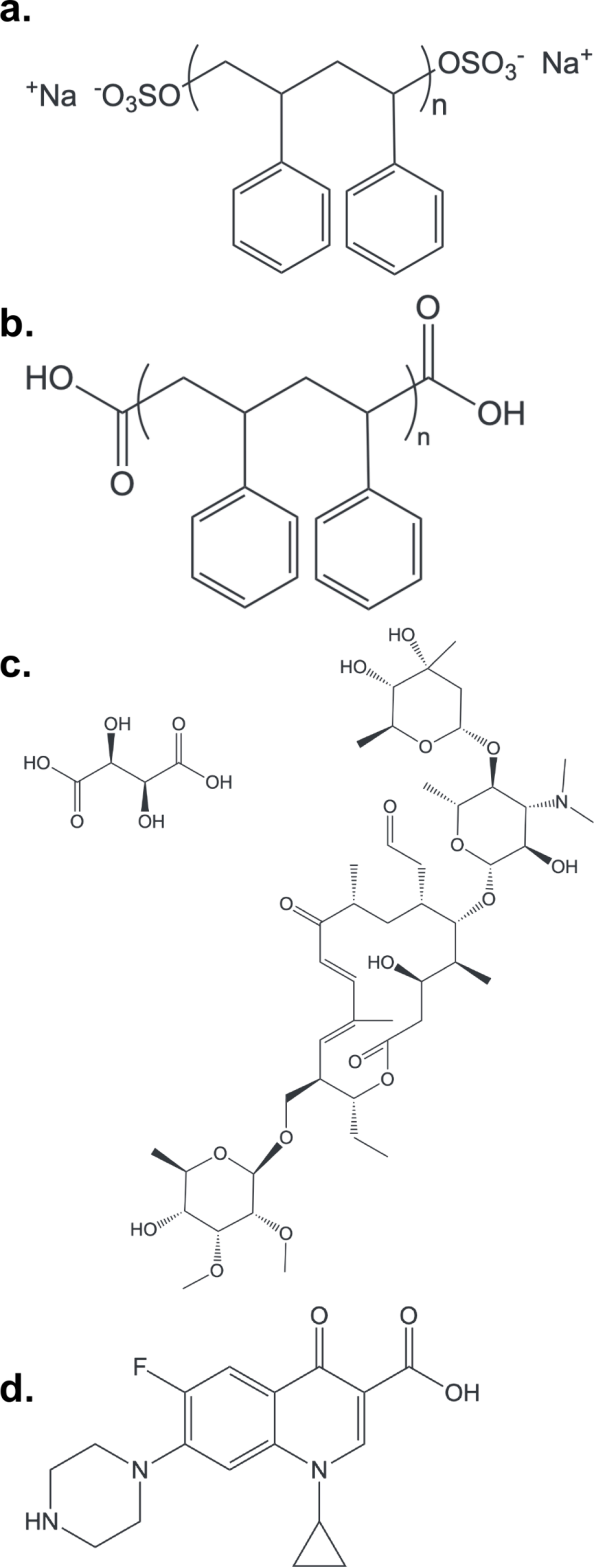
Chemical structures of particles. (a) Sulfate-terminated polystyrene nanoparticles (SO_4_-PSNPs) and (b) carboxyl-terminated polystyrene nanoparticles (COOH-PSNPs) and antibiotics (c) tylosin (TYL) tartrate and (d) ciprofloxacin (CIP) used in this study.

### Hydrodynamic and Electrokinetic Characterization of PSNPs

The apparent ζ potentials and hydrodynamic diameters of the PSNPs were determined using laser Doppler microelectrophoresis and dynamic light scattering (Malvern Zetasizer Nano ZS). Solutions were prepared at a PSNP concentration of 50 mg L^–1^ in either 1 or 100 mM NaCl buffered to pH 7.4 with 10 mM HEPES buffer solution (Sigma, 83264) and varying amounts of tylosin tartrate (Fisher Scientific, J62633) or ciprofloxacin hydrochloride monohydrate (Fisher Scientific, AAJ6197006). Structures of these two antibiotics are shown in Figure 1. Solutions were prepared by adding the appropriate amount of antibiotic to a 10 mM HEPES buffer of the desired ionic strength. PSNPs were added; the solution was vortexed, and the sample was analyzed. The following specifications were used for DLS measurements. The polystyrene latex material has a refractive index of 1.590 and an absorbance of 0.010. Five measurements were averaged together, and each measurement consisted of 10 runs where each run had a duration of 7 s. The diffusion coefficient of the particles was found using an intensity correlation function, which was then converted into a hydrodynamic diameter using the Stokes–Einstein equation. The hydrodynamic diameters reported are the *Z*-average values, representing an intensity-weighted mean hydrodynamic size of the particles. Additionally, polydispersity index values were found and are reported in the Supporting Information (Figure S2). For the ζ potential measurements, the temperature was allowed to equilibrate for 60 s, five measurements were averaged, and the measurement duration was set to a minimum of 10 runs and a maximum of 100 runs. All measurements were conducted at 25 °C.

### Interaction of PSNPs with Model Sediment Surfaces

We used QCM-D to monitor the amount of deposition of PSNPs of different terminal groups onto a model sediment surface, SiO_2_, in the presence and absence of an antibiotic. QCM-D measures changes in resonance frequency (Δ*f*) and dissipated energy (Δ*D*) as a function of time for a quartz crystal.

Before being exposed to the analyte, SiO_2_-coated QCM-D crystals (QSX303, Nanoscience Instruments) were cleaned by being sonicated in a 2% sodium dodecyl sulfate solution for 10 min, rinsed three times alternating between MQ water (18.2 MΩ cm resistivity, Millipore Synergy UV) and ethanol, and dried with N_2_ gas. The crystals were exposed to UV/ozone treatment for 10 min (Bioforce Nanosciences UV/Ozone Procleaner, 185 and 254 nm) and then immediately loaded into a temperature-controlled QSense flow module (QFM 401) on a QSense Explorer system (Biolin Scientific).

To quantify the mass of PSNPs deposited to a model sediment surface, the precleaned crystal was equilibrated in a buffer (10 mM HEPES (pH 7.4) with either 1 or 100 mM NaCl depending on the ionic strength of interest) at a flow rate of 0.100 mL min^–1^ until stable (defined as a Δ*f* of <0.3 Hz over 10 min). After equilibration, solutions of 50 mg L^–1^ PSNPs in HEPES buffer (pH 7.4) of the desired ionic strength and amount of antibiotic (resulting in an antibiotic-to-PSNP ratio of 0 mg_antibiotic_ mg_PSNPs_^–1^ or 0.4 mg_antibiotic_ mg_PSNPs_^–1^) were passed over the crystal. Once stable, the NaCl/buffer solution was passed over the crystal for 30 min or until stable to rinse away any unadsorbed or loosely bound particles from the surface. A schematic of this experimental protocol is shown in Figure S4.

For rigidly adsorbed films that met the criterion −Δ*D*_*n*_/(Δ*f*_*n*_/*n*) ≪ 2/*f_n_*, which for the crystals (4.96 MHz) used in this study is 4 × 10^–7^ Hz^–1^, the adsorbed surface mass density (Γ_QCM‑D_) is linearly proportional to the change in frequency (Δ*f*).^39^ All data in this manuscript meet this criterion, and as such, the Sauerbrey equation

1 $$\Gamma_{\mathrm{QCM‐D}}=-\frac{C}{n}\Delta f_{n}$$

where *C* is the mass sensitivity constant (17.7 ng cm^–2^ for a 5 MHz crystal) and *n* is the harmonic number, which in our case was the third harmonic, was used to relate the observed changes in frequency to changes in surface mass density in all cases. We probed the maximum amount of deposition as well as the reversibility of the deposition processes by also quantifying the final adsorbed surface mass density, which we recorded as the change in frequency between the end of the rinse and the stable baseline before the introduction of PSNPs. Frequency changes of <0.3 Hz (5 ng cm^–2^) were considered to be within noise and below the detection limit of the QCM-D and were not quantified as deposition in our study. All experiments were conducted in at least triplicate at 25.0 ± 0.5 °C.

### Statistical Analysis

To compare changes in hydrodynamic diameters, ζ potential values, and deposition, Student’s *t* statistical analyses were conducted using two-sample comparison of replicate measurement *t* tests assuming either equal or unequal variances at the α = 0.05 confidence level.

## Results and Discussion

### Motivation for Model System and Concentration Selections

It is important to note that the NPs used in this study, commercially available PSNPs, serve as a model to better understand the behavior of nanoplastics in a more controlled laboratory setting. However, “real nanoplastics”, defined here as nanosized plastic materials released as primary sources during manufacturing or resulting from the fragmentation or degradation of larger plastic materials in the environment, may be characterized as and behave differently compared to the particles used in our study. We refer to the particles used in our work as PSNPs to distinguish them from nanoplastics resulting from degradation or weathering processes. Previous work has shown that “real nanoplastics” may have rougher surfaces, increased surface areas, and the presence of additives and/or fillers, which can change their sorption properties.^40,41^ However, work using manufactured polystyrene nanoparticles, which are more well-defined and homogeneous, is still warranted. For example, these particles can be used to establish trends in behavior as a function of different environmental factors, such as ionic strength, temperature, pH, solvent conditions, and protein concentration,^42^ or strategically used to monitor the impact of different physiochemical properties of the nanomaterials (e.g., surface chemistry) on the environmental behavior of the nanomaterials.^43^ In our work, we chose these commercially available materials so that we could systematically test the role of surface chemistry, surface charge, ionic strength, and antibiotic concentration on PSNP–antibiotic and PSNP–sediment interactions.

The concentration of PSNPs used in this study was 50 mg L^–1^. While this concentration is higher than what is environmentally relevant for PSNPs,^44^ we chose this concentration in order to obtain detectable and reproducible signals for our ζ potential and hydrodynamic diameter measurements. Assuming an environmentally relevant estimate of the PSNP concentration^45^ of ∼2 μg L^–1^ and an environmentally relevant TYL concentration range^46^ of 0.04–0.28 μg L^–1^, we selected TYL:PSNP concentration ratios ranging from 0 to 0.4 (mg_TYL_ mg_PSNPs_^–1^) for our studies with TYL, corresponding to TYL:PSNP molar ratios ranging from 0 to 1.5 × 10^5^ (*M*_TYL_:*M*_PSNPs_). Similarly, CIP has been found environmentally^35^ at concentrations ranging from 0.01 to 1.5 μg L^–1^, and we used the same CIP:PSNP ratios ranging from 0 to 0.4 (mg_CIP_ mg_PSNPs_^–1^), corresponding to CIP:PSNP molar ratios ranging from 0 to 4 × 10^5^ (*M*_CIP_:*M*_PSNPs_) for our studies with CIP. Maintaining environmentally relevant concentration ratios allowed us to overcome the instrumental limitations imposed by the low environmentally relevant concentrations of the micropollutants we chose to study. We chose to analyze these systems at a pH of 7.4 in 1 mM NaCl, which both fall within the ranges of pH and ionic strength, respectively, encountered in natural freshwater systems.^47–49^ We also analyzed these interactions at an ionic strength of 100 mM NaCl, which is above that typically found in freshwater systems,^47^ to probe the role of electrostatics in the interactions (NP–antibiotic and NP–sediment) studied here.

### Impact of TYL on the Hydrodynamic and Electrokinetic Properties of PSNPs

Panels a and b of Figure 2 (blue circles) show the impact of an increasing TYL concentration on the hydrodynamic and ζ potential properties of the SO_4_-PSNPs in 10 mM HEPES and 1 mM NaCl. Initially, the hydrodynamic diameter of the SO_4_-PSNPs is 114 ± 2 nm. As the TYL:SO_4_-PSNP concentration ratio increases from 0 to 0.4 mg_TYL_ mg_SO_4_‑PSNPs_^–1^, there is no observable change in the hydrodynamic diameter of SO_4_-PSNPs (110 ± 1 nm (Figure 2a)). In contrast, there is a significant decrease (*n* = 5, α = 0.05) in the ζ potential of SO_4_-PSNPs from 0 to 0.4 mg_TYL_ mg_SO_4_‑PSNPs_^–1^, shifting from −30 ± 1 to −37 ± 1 mV, respectively (Figure 2b, blue circles), and a decrease in the polydispersity index (from 0.06 ± 0.01 to 0.02 ± 0.01 (Figure S2a)), suggesting a slight electrostatic stabilizing effect of the TYL at 1 mM NaCl and a slightly more homogeneous sample. At 100 mM NaCl, the addition of TYL, at a mg_TYL_:mg_SO_4_‑PSNPs_ ratio of ≥0.04, results in hydrodynamic diameters of greater than 1000 nm and polydispersity index values ranging from 0.1 to 0.8, suggesting that TYL induces aggregation of the SO_4_-PSNPs (Figures 2a, orange circles, and Figure S2a). The ζ potential value significantly increases (*n* = 5, α = 0.05) from −31 ± 1 to −7 ± 1 mV as the mg_TYL_:mg_SO_4_‑PSNPs_ concentration ratio increases from 0 to 0.4, respectively (Figure 2b, orange circles). The low-magnitude ζ potential at 100 mM NaCl and a high TYL:SO_4_-PSNP concentration ratio cause attractive van der Waals forces between the particles to overcome the electrostatic repulsive forces and result in the observed aggregation. Previous work has attributed the sorption of TYL onto 3 μm polystyrene microparticles to a combination of electrostatic interactions, surface complexation, and hydrophobic interactions,^50^ all of which are also likely involved in our observed sorption of TYL to the smaller 100 nm polystyrene particles used in this work. Charge neutralization has previously been shown to induce aggregation of NPs.^23,51^ In particular, cationic polymers have been shown to induce aggregation with negatively charged SO_4_-PSNPs,^52,53^ which supports our observations and suggests a favorable interaction between the negatively charged sulfate groups on the surface of the SO_4_-PSNPs and the positively charged amine groups in TYL.

**Figure 2:**
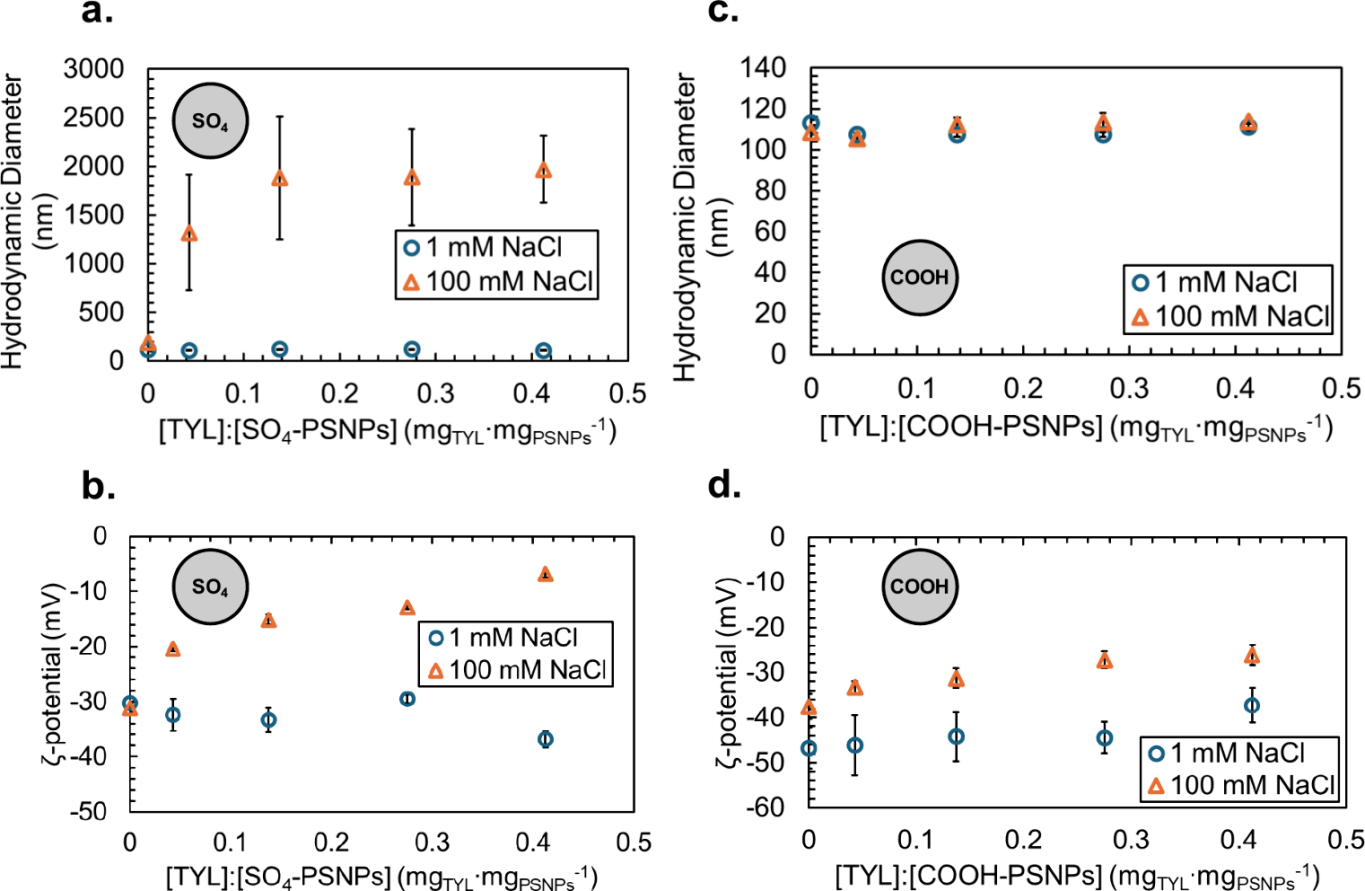
Impact of an increase in the TYL concentration on the (a and c) hydrodynamic diameter and (b and d) ζ potential of (a and b) SO_4_-PSNPs and (c and d) COOH-PSNPs in 10 mM HEPES buffered to pH 7.4 with either 1 mM NaCl (blue circles) or 100 mM NaCl (orange triangles). Error bars represent the standard deviation of five replicate measurements.

To determine whether the interactions between TYL and the SO_4_-PSNPs were driven based on surface chemistry or surface charge, we conducted a similar study using negatively charged PSNPs with carboxyl group termination, COOH-PSNPs (Figure 1). In 10 mM HEPES with 1 mM NaCl and no TYL present, the COOH-PSNPs had a hydrodynamic diameter of 113 ± 2 nm, a ζ potential of −47 ± 5 mV (Figure 2c,d, blue circles), and a polydispersity index of 0.06 ± 0.01 (Figure S2c), compared to a hydrodynamic diameter of 114 ± 2 nm and a ζ potential of −30 ± 1 mV for SO_4_-PSNPs in the absence of TYL (Figure 2a,b, blue circles). When increasing amounts of TYL were added to the COOH-PSNPs, there was no change in the hydrodynamic diameter in 10 mM HEPES and 1 mM NaCl (Figure 2b, blue triangles), with the size remaining constant at 111 ± 2 nm for a mg_TYL_:mg_COOH‑PSNPs_ concentration ratio of 0.4, or in the polydispersity index (0.05 ± 0.01 (Figure S2c)). The ζ potential of COOH-PSNPs is significantly higher (*n* = 5, α = 0.05) with a mg_TYL_:mg_COOH‑PSNPs_ concentration ratio of 0.4, −37 ± 4 mV, than when in the absence of TYL, −47 ± 5 mV, at 1 mM NaCl (Figure 2d, blue circles). At 100 mM NaCl, the COOH-PSNPs have a hydrodynamic diameter of 109 ± 1 nm and do not aggregate following the addition of TYL with a hydrodynamic diameter of 114 ± 1 nm (Figure 2c, orange triangles) at a mg_TYL_:mg_COOH‑PSNPs_ concentration ratio of 0.4. However, the ζ potential of COOH-PSNPs does significantly increase (*n* = 5, α = 0.05) from −38 ± 3 to −26 ± 2 mV (Figure 2d, orange triangles) as the mg_TYL_:mg_COOH‑PSNPs_ concentration ratio increases from 0 to 0.4. Additionally, the polydispersity index significantly increases from 0.06 ± 0.01 to 0.08 ± 0.01 (*n* = 5, α = 0.05 (Figure S2c)) in the presence of TYL, suggesting a more heterogeneous sample upon the addition of TYL. The relatively high magnitude of the negative ζ potential at 100 mM NaCl and 0.4 mg_TYL_:mg_COOH‑PSNPs_ (−26 ± 2 mV) results in electrostatic repulsions and a lack of observed aggregation for the COOH-PSNPs. The observed differences in the changes in aggregation state and ζ potential upon the addition of TYL to COOH-PSNPs compared to SO_4_-PSNPs suggest surface chemistry plays a larger role in dictating these interactions compared to the initial surface charge as both the COOH-PSNPs (−38 ± 3 mV) and the SO_4_-PSNPs (−31 ± 1 mV) had similar starting ζ potentials. Previous work showed that unfunctionalized PSNPs behaved differently than COOH-functionalized PSNPs in terms of the sorption of two fluoroquinolones, with COOH-functionalized particles having a much higher sorption capacity.^31^ For TYL, in particular, previous work has suggested that its sorption to micrometer-sized polystyrene is due to a combination of electrostatic and hydrophobic interactions.^50^ These findings support our results that the surface chemistry of the NPs plays a larger role in the sorption of copollutants than charge alone, with the addition of TYL leading to the aggregation of the SO_4_-PSNPs as compared to the COOH-PSNPs likely due to a complex interplay between electrostatic and hydrophobic interactions.

The TYL used in this study was in the form of tylosin tartrate, meaning that free tartrate is in solution. To verify that our observed changes were due to TYL and not free tartrate in solution, we conducted control experiments to investigate the impacts of tartrate on the hydrodynamic diameter and ζ potential of both COOH-PSNPs and SO_4_-PSNPs at the tartrate:PSNP concentration ratios that would be observed at our selected TYL:PSNP concentration ratios. We observed no changes due to the presence of tartrate that could explain the observations seen in Figure 2, suggesting the observed impacts on the physiochemical properties of the two types of PSNPs in Figure 2 are due to the presence of TYL, not the negatively charged free tartrate in solution (Figure S3).

### Impact of CIP on the Hydrodynamic and Electrokinetic Properties of PSNPs

We compared our TYL results with those of a second model antibiotic, CIP (Figure 1). We chose CIP due to its zwitterionic nature at a pH of 7.4. At 1 mM NaCl, we observed no significant changes (*n* = 5, α = 0.05) in hydrodynamic diameter for the SO_4_-PSNPs (Figure 3a, blue circles; 113 ± 2 to 114 ± 4 nm from 0 to 0.4 mg_CIP_:mg_SO_4_‑PSNPs_, respectively) or the COOH-PSNPs (Figure 3c, blue circles; 109 ± 4 to 105 ± 2 nm from 0 to 0.4 mg_CIP_:mg_COOH‑PSNPs_, respectively) following exposure to CIP. The polydispersity indices also remained relatively constant for both particle types at the low ionic strength (Figure S2b,d). Similarly, we observed no significant changes (*n* = 5, α = 0.05) in ζ potential for the SO_4_-PSNPs (Figure 3b, blue circles; −39 ± 2 to −36 ± 4 mV from 0 to 0.4 mg_CIP_:mg_SO_4_‑PSNPs_, respectively) or the COOH-PSNPs (Figure 3d, blue circles; −54 ± 7 to −49 ± 4 mV from 0 to 0.4 mg_CIP_:mg_COOH‑PSNPs_, respectively) following exposure to CIP at 1 mM NaCl. At 100 mM NaCl, we observed no significant changes (*n* = 5, α = 0.05), in hydrodynamic diameter for the SO_4_-PSNPs (Figure 3a, blue circles; 110 ± 2 to 112 ± 7 nm from 0 to 0.4 mg_CIP_:mg_SO_4_‑PSNPs_, respectively) and a slight decrease in size (*n* = 5, α = 0.05) for the COOH-PSNPs (Figure 3c, orange triangles; 106 ± 1 to 102 ± 1 nm from 0 to 0.4 mg_CIP_:mg_COOH‑PSNPs_, respectively) following exposure to CIP. Additionally, both showed no significant increases in polydispersity index values (0.03 ± 0.01 to 0.04 ± 0.03 for COOH-PSNPs and 0.04 ± 0.03 to 0.08 ± 0.04 for SO_4_-PSNPs (Figure S2b,d)), suggesting no changes to the polydispersity upon the addition of CIP. However, at 100 mM NaCl, a significant difference in ζ potential for both the SO_4_-PSNPs (Figure 3b, orange triangles; −38 ± 1 to −22 ± 1 mV from 0 to 0.4 mg_CIP_:mg_SO_4_‑PSNPs_, respectively) and the COOH-PSNPs (Figure 3d, orange triangles; −43 ± 2 to −38 ± 1 mV from 0 to 0.4 mg_CIP_:mg_COOH‑PSNPs_, respectively) was observed following exposure to CIP.

**Figure 3:**
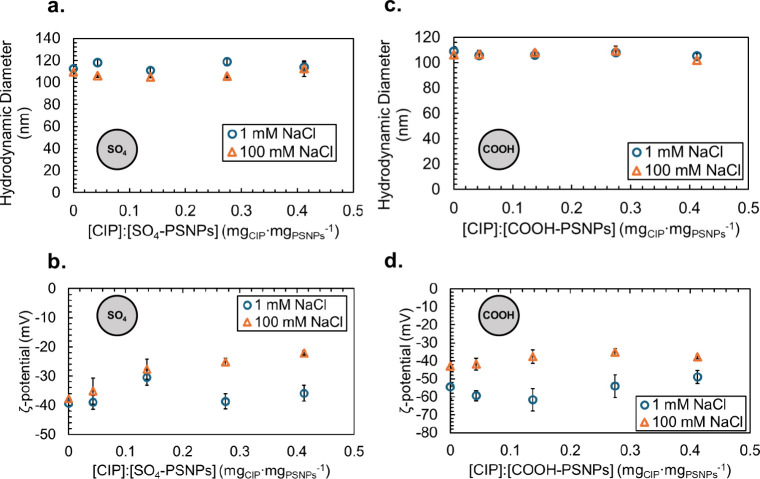
Impact of an increase in CIP concentration on the (a and c) hydrodynamic diameter and (b and d) ζ potential of (a and b) SO_4_-PSNPs and (c and d) COOH-PSNPs in 10 mM HEPES buffered to pH 7.4 with either 1 mM NaCl (blue circles) or 100 mM NaCl (orange triangles). Error bars represent the standard deviation of five replicate measurements.

Similar to TYL, an increase in ionic strength (1 to 100 mM NaCl) resulted in an increase in the adsorption of CIP. We hypothesize this is due to an increase in van der Waals attractive forces. Studies have previously found that CIP will adsorb strongly to polystyrene microplastics.^54^ Li et al. attribute the attraction of CIP to the polystyrene microplastics to van der Waals forces and π–π interactions between the aromatic portions of polystyrene and CIP.^54^ While the PSNPs used in our study are on the nanoscale, we hypothesize that similar interactions are occurring in our system. In addition, hydrogen bonding could occur between the deprotonated carboxylate groups on COOH-PSNPs and the amine group on CIP. Li et al. identify strong hydrogen bonding between a polyamide microplastic and the carboxylate on CIP.^54^ Since a smaller amount of interaction was observed with the COOH-PSNPs than with the SO_4_-PSNPs, we hypothesize the charge screening of the PSNPs and corresponding van der Waals forces have a larger effect than hydrogen bonding. Although nonelectrostatic forces are the main mechanism by which CIP adsorbs at pH 7.4, CIP can also have electrostatic interactions. Yilimulati et al. determined that the adsorption of CIP to COOH-PSNPs decreases as pH increases.^55^ As CIP converts from a positive to a zwitterionic and finally a negative molecule, the electrostatic attraction between CIP and negatively charged COOH-PSNPs decreases. At pH 7.4, the charge interactions are neither purely attractive nor repulsive, and we see that nonelectrostatic interaction takes precedence. CIP is more hydrophilic than TYL with a logK_ow_ of 0.28,^35^ so unlike TYL, hydrophobic interactions are not a likely cause of adsorption. Therefore, the adsorption of CIP to the PSNPs at pH 7.4 is likely governed by a combination of van der Waals attraction, π–π interactions, and hydrogen bonding more so than electrostatic or hydrophobic interactions.

### Comparison of *K*_a_ Values for the Binding of TYL or CIP to SO_4_-PSNPs

Based on the observed ζ potential and hydrodynamic diameter changes to the PSNPs in the presence of antibiotics, we wanted to further elucidate information about the binding processes occurring in our systems. However, determination of binding constants for charged antibiotics interacting with charged nanoparticles is nontrivial. Often Langmuir adsorption isotherms can be fit by using experimental data collected from a range of different techniques. UV–vis spectroscopy has been used to determine binding constants for proteins binding to plasmonic nanomaterials;^56,57^ changes in hydrodynamic diameter have been used to discern binding constants when agglomeration is not observed,^56,57^ and fluorescence spectroscopy has been used when the ligand of interest is intrinsically fluorescent.^57,58^ The extension of these methods to determine *K*_a_ values on charged antibiotics binding to polystyrene nanoparticles has been much less studied in the field. Recent studies relied on the use of radiolabeled compounds,^59^ saturation-transfer difference NMR,^60^ and computational approaches^61^ to elucidate information about the binding of antibiotics to polystyrene nanoparticles.

For the systems used in our work, the PSNPs do not have a plasmon band, the antibiotics of interest are not inherently fluorescent, and in the case of CIP and SO_4_-PSNPs there were no changes in hydrodynamic diameter observed when the CIP concentration was increased. Taken together, these three points complicated our efforts to quantify the observed binding processes. As an estimate to quantify the differences in interactions that we observed between the SO_4_-PSNPs in the presence of TYL compared to CIP at 100 mM NaCl, where changes in ζ potential were observed in both cases as the concentration of the antibiotic increased, we used a Langmuir adsorption isotherm fit with the ζ potential data according to eq 2:

2 $$\frac{\Delta \zeta }{\Delta \zeta_{\max}}=\frac{K_{a}C_{\mathrm{ant}}}{1+K_{a}C_{\mathrm{ant}}}$$

where Δζ and Δζ_max_ are the shift and maximum shift, respectively, in the ζ potential values, *K*_a_ is the binding constant (M^–1^), and *C*_ant_ is the antibiotic concentration (M). The Langmuir adsorption isotherms for SO_4_-PSNPs with TYL and for SO_4_-PSNPs with CIP at 100 mM NaCl are shown in Figure 4. From these approximations, we found a *K*_a_ value for the adsorption of CIP on SO_4_-PSNPs of 7.7 × 10^4^ M^–1^ and a *K*_a_ for the adsorption of TYL on SO_4_-PSNPs that was an order of magnitude higher with a value of 3.1 × 10^5^ M^–1^. This suggests that the interactions of TYL with SO_4_-PSNPs are stronger than those of CIP with SO_4_-PSNPs. This is supportive of the magnitude of changes we observed in the hydrodynamic diameter and ζ potential in the presence of TYL (Figure 2a,b) compared to CIP (Figure 3a,b).

**Figure 4:**
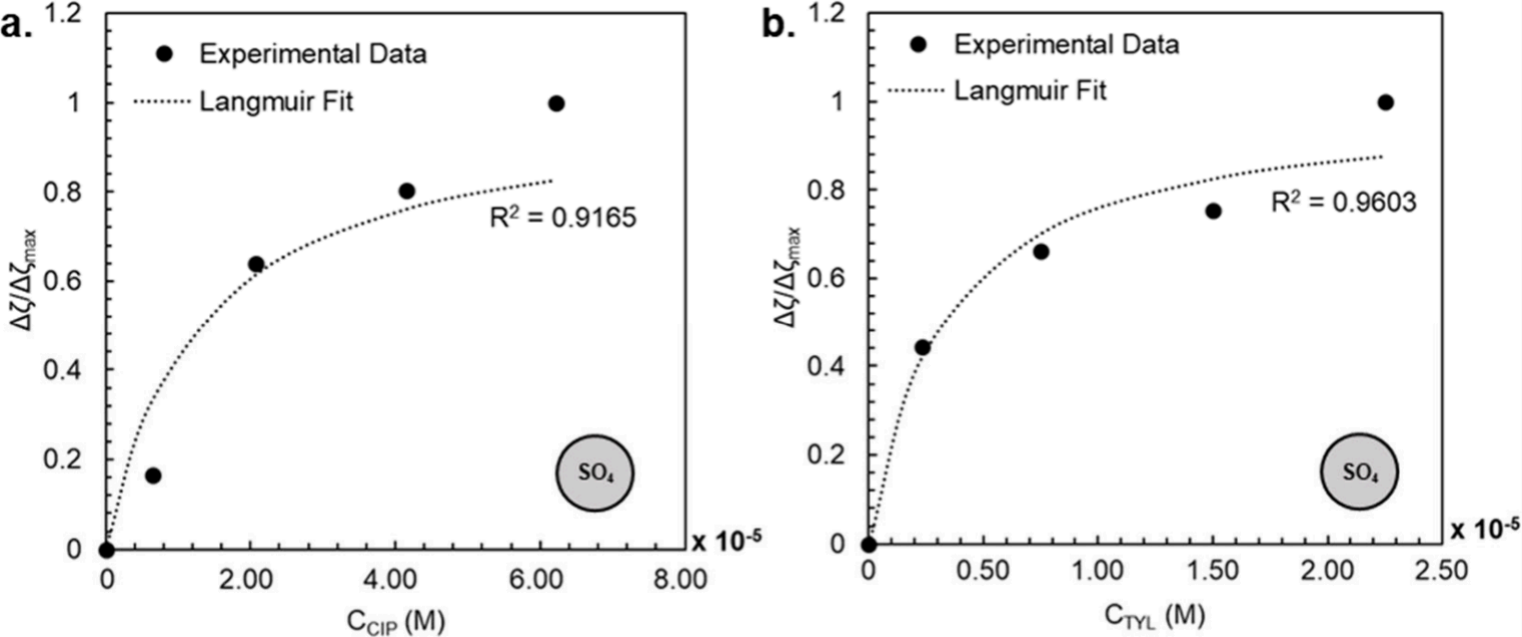
Langmuir adsorption isotherms for the binding of (a) CIP and (b) TYL to SO_4_-PSNPs in 100 mM NaCl. Both of these conditions showed a change in ζ potential as a function of antibiotic concentration, which allowed us to fit this relationship to eq 2 (dashed lines).

Fitting these data sets to a Langmuir adsorption isotherm assumes that every binding site is identical, there are no interactions between adsorbed molecules, there is only a monolayer of adsorption,^62^ and the ζ potential changes proportionally with surface coverage. While these assumptions may not be wholly true in the case of our systems, this approach provided an estimation of *K*_a_ values that allowed us to compare the two data sets to obtain relative binding constant information. Approaches relating ζ potential to adsorption have been demonstrated in the literature for applications similar to ours such as the adsorption of cobalt ions on superparamagnetic nanoparticles,^63^ the adsorption of charged drugs on phospholipid vesicles,^64^ the adsorption of ions on goethite,^65^ and the adsorption of surfactants on coal.^66^

### Deposition of PSNPs onto SiO_2_ in the Presence and Absence of Antibiotics

Knowing that TYL and CIP change the physiochemical properties of PSNPs, we wanted to understand how these transformations may impact the subsequent environmental behavior of the PSNPs. While previous studies have used QCM-D to quantify the deposition of NPs in the presence of other organic species, these studies have mainly focused on the impact of natural organic matter^23,24,67^ on the deposition of NPs onto environmental surfaces rather than the presence of copollutants, such as antibiotics. Here, we used QCM-D to monitor the deposition of PSNPs (COOH-PSNPs or SO_4_-PSNPs) onto a SiO_2_-coated quartz crystal, intended as a model sediment surface. We controlled the water chemistry by including other organic pollutants (e.g., TYL or CIP) and NaCl (1 or 100 mM) to control the ionic strength. We selected SiO_2_ as our model sediment surface because it is one of the major components of dissolved solids found in aquatic systems.^68^ In each case, we looked at the deposition of the PSNPs in the absence of antibiotics, the deposition of the antibiotics in the absence of PSNPs, and a combination of each type of PSNPs with each antibiotic at a concentration ratio of 0.4 mg_antibiotic_:mg_PSNPs_, where differences in the physiochemical properties of the PSNPs were observed.

At 1 mM NaCl, very little deposition of the micropollutants onto the SiO_2_ crystal was detected (Table S1). The COOH-PSNPs, the SO_4_-PSNPs, and TYL independently all showed no quantifiable deposition, both before and after rinsing. CIP produced quantifiable initial deposition, 29 ± 17 ng cm^–2^ (Table S1), but upon rinsing, it was removed from the SiO_2_ surface, suggesting a reversible interaction. At 1 mM NaCl with 0.4 mg_TYL_:mg_PSNPs_, the SO_4_-PSNPs and COOH-PSNPs also showed no deposition. However, like CIP alone, both PSNPs in combination with CIP showed initial deposition, indistinguishable from CIP alone, and substantial removal upon rinsing (Table S1), likely suggesting attachment of free CIP in solution to the crystal that was then rinsed away. The SiO_2_ crystals used in this study are known to have a ζ value of −98 ± 2 mV in 10 mM HEPES and 10 mM NaCl,^69^ which falls between the ionic strengths of the buffers used in this study. Electrostatic repulsions are likely between the negatively charged PSNPs and the negatively charged SiO_2_ substrate. In addition, the SiO_2_ surface is hydrophilic, which may repulse the more hydrophobic antibiotics.^70^

At 100 mM NaCl, both the COOH-PSNPs (initial, 66 ± 3 ng cm^–2^; rinsed, 31 ± 13 ng cm^–2^) and the SO_4_-PSNPs (initial, 190 ± 80 ng cm^–2^; rinsed, 170 ± 40 ng cm^–2^) deposited onto the SiO_2_ surface, with some removal upon rinsing (Table S1 and Figure 5). At 1 mM NaCl, the unfavorable charge interaction between the negatively charged PSNPs and the SiO_2_ surface inhibited the deposition of PSNPs. However, at 100 mM NaCl, more ions screen the negative charges of the PSNPs and SiO_2_, and the van der Waals attractive forces between the PSNPs and SiO_2_ surface are more prominent. Previous studies have shown the deposition of negatively charged 50 nm PSNPs onto a SiO_2_ crystal increased at higher NaCl concentrations, which the authors attributed to charge screening and decreased electrostatic repulsion.^22^ The surface functionalization of the PSNPs did affect the amount of deposition. The SO_4_-PSNPs deposited significantly more (*n* = 4, α = 0.05) onto the SiO_2_ surface than the COOH-PSNPs (170 ± 40 and 31 ± 13 ng cm^–2^, respectively) (Figure 5 and Table S1). Previous studies revealed larger amounts of deposition of sulfate-functionalized polystyrene compared to carboxyl-modified polystyrene onto aluminum oxide. The authors attributed the difference in their work to the hydrophobicity differences between the carboxyl and sulfate groups and associated the higher deposition rate of the sulfate particles compared to the carboxyl particles with the hydrophobic effect,^25^ which may also explain our observations. The increased size and lower charge density of sulfonate groups compared to carboxyl groups have been used in the literature about per- and polyfluoroalkyl substances (PFAS) to explain greater deposition of perfluorooctanesulfonate (PFOS) than perfluorooctanoic acid (PFOA) onto negatively charged biological surfaces.^71^ This could be relevant to our studies, as well, with the increased deposition of SO_4_-PSNPs compared to COOH-PSNPs on our model sediment surface being in part due to the increased size and lower charge density of the sulfate groups on the PSNPs compared to the carboxyl groups on the PSNPs.

**Figure 5:**
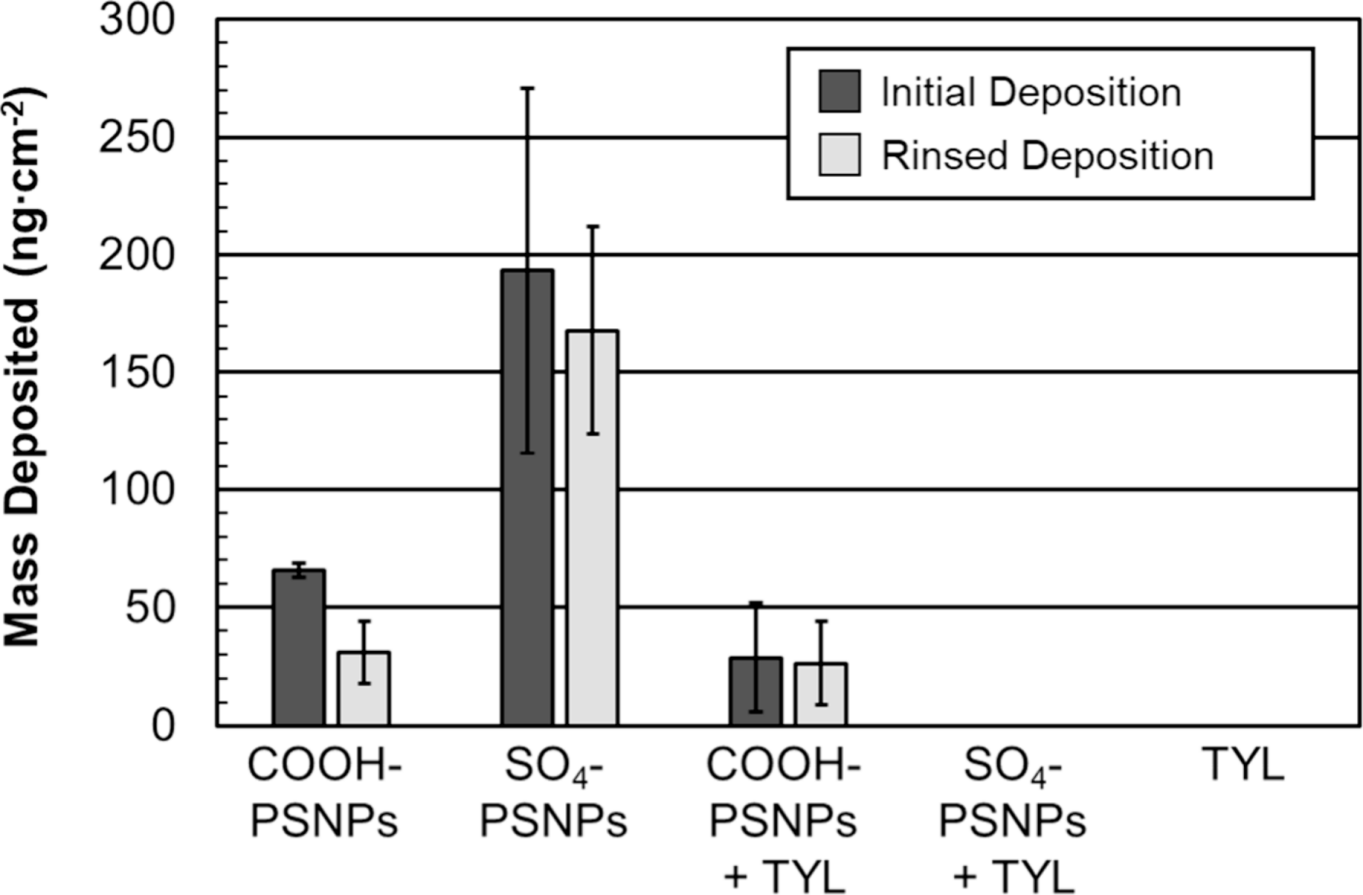
Initial mass deposited (ng cm^–2^, dark gray) and mass deposited (ng cm^–2^, light gray) following a buffer rinse of the indicated micropollutants in 100 mM NaCl. The concentration ratio when TYL and PSNPs are present is 0.4 TYL:PSNP (mg_TYL_:mg_PSNPs_), with just PSNPs 50 mg L^–1^, and with just TYL 20 mg L^–1^. The error bars are standard deviations for at least three trials.

The presence of TYL and its high *K*_a_ value for SO_4_-PSNPs diminished the attachment of the SO_4_-PSNPs to the SiO_2_ surface in 100 mM NaCl buffer solutions (Figure 5 and Table S1). At a pH of 7.4, some of the amine groups of TYL will be protonated. Based solely on electrostatics, we hypothesized that the positively charged amine groups on TYL would interact more favorably with the negatively charged silica than the bare negatively charged SO_4_-PSNPs. However, the opposite was observed. The larger aggregates formed at 0.4 mg_TYL_:mg_PSNPs_ concentration ratios (Figure 2a) result in less deposition. At 100 mM NaCl, the diameter of the SO_4_-PSNPs/TYL aggregates is ∼150 times the size of SO_4_-PSNPs alone (Figure 2a). Quevedo et al. determined that particle aggregation can decrease the deposition of species in QCM-D because larger particles experience slower diffusion^72^ and less convective-diffusive particle transport^25^ to the underlying SiO_2_ sediment. Liu et al. also determined that larger PSNPs (500 nm) will have weaker attachment to silica than smaller PSNPs (50 nm) based on Derjaguin–Landau–Verwey–Overbeek (DLVO) theory.^22^ When aggregated, the bulk transport and flow of the solution impact the movement of the particles more than diffusive motion that promotes interaction between the surface and particles; thus, it is likely that the SO_4_-PSNPs/TYL complex is swept away before it can interact with the SiO_2_ surface. As a result, no deposition is observed with the SO_4_-PSNPs/TYL complex despite TYL making SO_4_-PSNPs less negatively charged.

TYL did not have a significant effect on the deposition of COOH-PSNPs in 100 mM NaCl (Figure 5 and Table S1). When TYL is combined with COOH-PSNPs at 100 mM NaCl, the ζ potential of the complex remains quite negatively charged (Figure 2d) and the size remains relatively constant (Figure 2b). Based on the DLS and ζ potential results, it is likely that there is minimal interaction between the TYL and the COOH-PSNPs. This is confirmed with the QCM-D results where TYL alone shows no interaction with the SiO_2_ surface (Table S1 and Figure 5) and the COOH-PSNPs alone (31 ± 13 ng cm^–2^) show comparable deposition to the combination of COOH-PSNPs with TYL (26 ± 18 ng cm^–2^ (Table S1 and Figure 5)).

Initial deposition of the COOH-PSNPs in the presence of CIP (180 ± 40 ng cm^–2^), prior to rinsing, at 100 mM NaCl is significantly higher than deposition of the COOH-PSNPs alone (66 ± 3 ng cm^–2^) prior to rinsing. However, upon rinsing, no significant difference in the depositions is observed (Figure 6 and Table S1). Similarly, the initial deposition of the SO_4_-PSNPs in the presence of CIP (400 ± 140 ng cm^–2^) is significantly higher than deposition of SO_4_-PSNPs alone (190 ± 80 ng cm^–2^). However, upon rinsing, no significant difference in their depositions is observed (Figure 6 and Table S1). Despite the deposition of CIP alone not being detected, its presence does increase the ζ potential of both particle types. This charge screening effect likely explains the observation that the CIP promotes the attachment of both types of PSNPs to the underlying SiO_2_ surface by decreasing charge repulsion. However, the loosely adhered CIP between particles appears to be removed upon rinsing, making the behavior of the PSNPs similar following rinsing with or without CIP.

**Figure 6:**
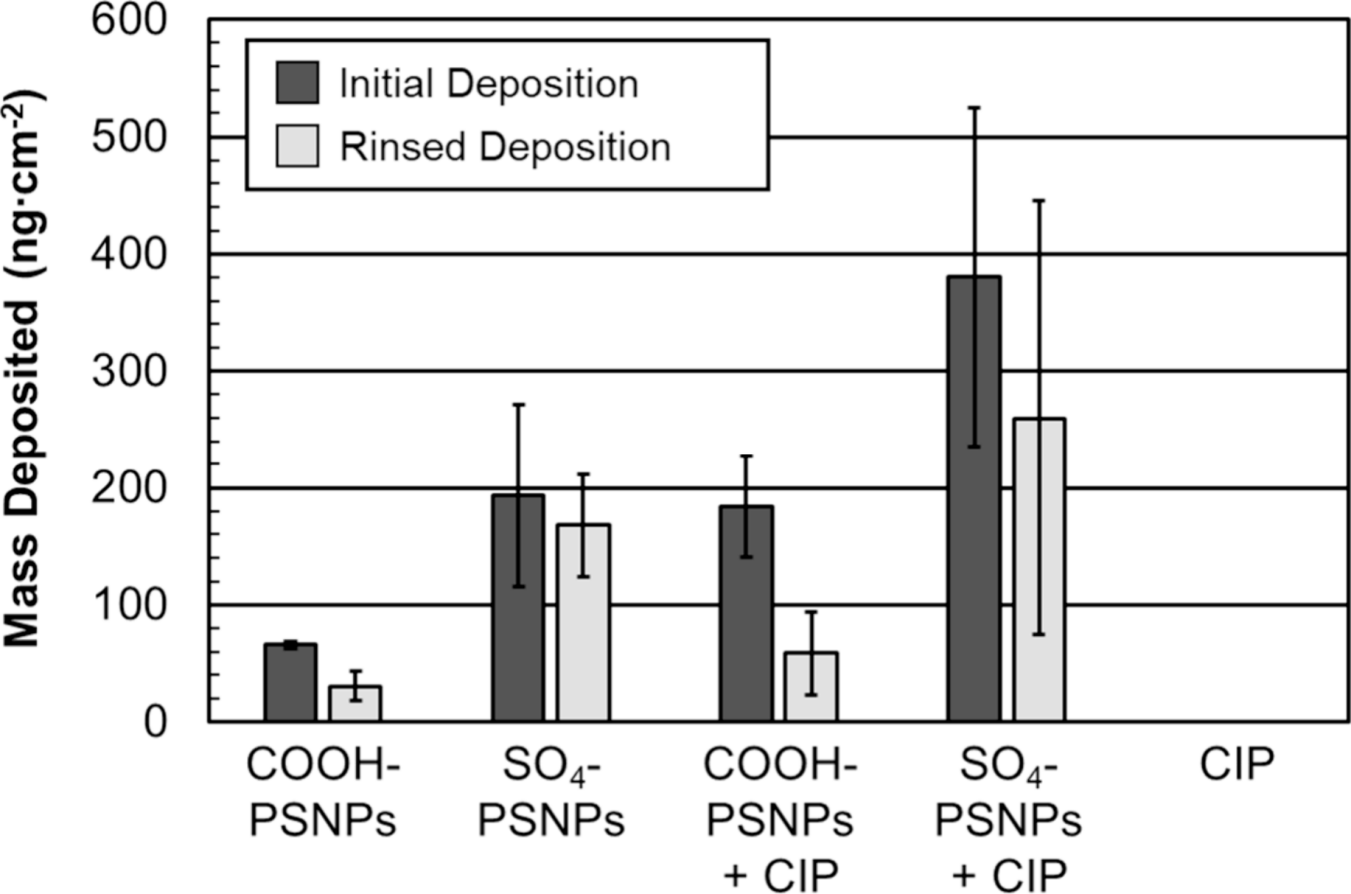
Initial mass deposited (ng cm^–2^, dark gray) and mass deposited (ng cm^–2^, light gray) following a buffer rinse of the indicated micropollutants in 100 mM NaCl. The concentration ratio when CIP and PSNPs are present is 0.4 CIP:PSNPs (mg_CIP_:mg_PSNPs_), with just PSNPs 50 mg L^–1^, and with just CIP 20 mg L^–1^. The error bars are standard deviations for at least three trials.

## Conclusions

The combined properties of pollutants are different than the sum of the properties of pollutants alone. As the complexity of waste and aqueous species increases, the impact of multipollutant interactions will dictate how pollutants move through natural systems and how the pollutants interact with and impact the environment. Here, the interactions between two groups of common pollutants, NPs and antibiotics, were probed as model systems.

The interactions governing the adsorption of the antibiotics to the NPs and the interactions between the model surface and the combined NP/antibiotic complexes likely exist for other systems. Our work highlights the complex interplay among the NP surface functionalization and charge, the total ionic strength of the solution, and the hydrophilicity and charge of the NPs and pollutant in dictating the NP/pollutant behavior. While the adsorption of antibiotics to PSNPs is likely due to hydrophobic effects and van der Waals forces, deposition onto the SiO_2_ surface depends more on how charge screening impacts the charge of the surface and particles.

At higher ionic strengths, favorable electrostatic forces between CIP and the SiO_2_ surface were weakened due to charge screening, resulting in less CIP deposition onto the SiO_2_ surface. For PSNPs, a higher ionic strength weakened the repulsive electrostatic forces, yielding an increased deposition of PSNPs onto SiO_2_ via van der Waals interactions. When combined, the formation of the less negatively charged PSNPs/CIP complex enhanced the deposition of both onto SiO_2_ at 100 mM NaCl. The van der Waals interactions between antibiotics and PSNPs and between the PSNPs/antibiotic complexes and the SiO_2_ surface increase with ionic strength. Meanwhile, the formation of SO_4_-PSNPs/TYL aggregates prevented the deposition of both at 100 mM NaCl because of the slow diffusion of the aggregates. Schematics summarizing our findings and the modes of deposition of the PSNPs alone or in the presence of antibiotics onto the underlying SiO_2_ substrate can be found in Figure S5.

Examining multipollutant systems requires a full understanding of the forces working between pollutants, forces between the sediment and pollutants separately, and the possible combined forces of the pollutants and sediment surface. Because of this, studies often focus on only a single pollutant. Due to the complexity of natural waters, more research on how multipollutant systems behave is needed as single-pollutant systems may not fully explain how pollutants partition in the environment. Additional work is also needed to increase the complexity of the water chemistries studied. Here we focused on systems containing Na^+^ and Cl^–^ ions to represent the ionic strengths of natural waters and probe the role of electrostatics in these interactions (PSNP–antibiotic and PSNP–sediment); however, the composition of natural waters includes other constituents such as divalent cations, natural organic matter, and heavy metals, which may also impact the behavior of nanomaterials, and future studies aimed at increasing the water chemistry complexity are warranted. In addition, the use of weathered and fragmented nanoplastics may show different results than those obtained on the manufactured PSNPs used here. Previous work, conducted on polystyrene microparticles, has shown that the pure polymer microparticles had lower uptake of micropollutants (such as PFAs, atrazine, and acetamidophenol) than real microparticles in most cases.^40^ This highlights a need for nanosized model systems representative of real nanoplastics, but the detection and recovery of nanosized, carbon-based materials in natural waters present methodological challenges and complicate the feasibility of these types of studies.^4^ Ultimately, our results cannot be generalized to describe the behavior of all nanoplastics in all water chemistries but instead represent findings that can guide the design of future studies to better understand the behavior of “real nanoplastics” in more complex natural waters.

## Supplementary Material



## Notes

Scanning electron microscopy images of the particles used in this study, DLS and ζ potential control experiments investigating interactions of tartrate with PSNPs, a schematic showing the experimental protocol of the QCM-D experiments, and a table summarizing the QCM-D results (PDF)

## #

A.L.D.: Department of Chemistry, University of Michigan, Ann Arbor, MI 48109

## ⊥

E.A.G.: U.S. Environmental Protection Agency Office of Air Quality Planning and Standards, Research Triangle Park, NC 27711

## Abbreviations

COOH-PSNPs: carboxyl-terminated polystyrene nanoparticles
SO_4_-PSNPs: sulfate-terminated polystyrene nanoparticles
TYL: tylosin
CIP: ciprofloxacin
QCM-D: quartz crystal microbalance with dissipation monitoring
