# Supplementary material for: Impact of Tylosin Tartrate and Ciprofloxacin on the Deposition of Negatively Charged Polystyrene Nanoparticles onto SiO2
Source: Langmuir. 2025 Jul 15;41(29):19183–93. doi: 10.1021/acs.langmuir.5c01405 (PMC12312160; doi:10.1021/acs.langmuir.5c01405)
Supplement: Supplementary file 1 [file la5c01405_si_001.pdf]

Supporting Information for:

Impact of tylosin tartrate and ciprofloxacin on the deposition of negatively charged polystyrene nanoparticles to SiO_2

Anna L. DiFelice[†,#], *Anna Silver*[†], *Elizabeth A. Good*[†,⊥], *Arielle C. Mensch*[†,*]

AUTHOR ADDRESS

[†]Department of Chemistry, Lafayette College, Easton, PA 18042

[#]Department of Chemistry, University of Michigan, Ann Arbor, MI 48109

[⊥]U.S. Environmental Protection Agency Office of Air Quality Planning and Standards, Research Triangle Park, NC 27711.

Contents

Supplemental Figures

Figure S1. Scanning electron micrographs of the SO_4-PSNPs and COOH-PSNPs **S3**

Figure S2. Polydispersity index values for the PSNPs as a function of [TYL] or [CIP] **S4**

Figure S3. Hydrodynamic and electrokinetic characterization of SO_4-PSNPs and COOH-PSNPs as a function of [tartrate]:[PSNP] ratio **S5**

Figure S4. Schematic showing the work flow of the QCM-D experiments **S6**

Figure S5. Schematic showing the hypothesized modes of deposition for COOH-PSNPs, COOH-PSNPs + TYL, COOH-PSNPs + CIP, SO_4-PSNPs, SO_4-PSNPs + TYL, SO_4-PSNPs + CIP **S7**

Supplemental Tables

Table S1. Summary of mass deposited (ng·cm^2) prior to and after rinse with buffer for each of the pollutants studied **S8**

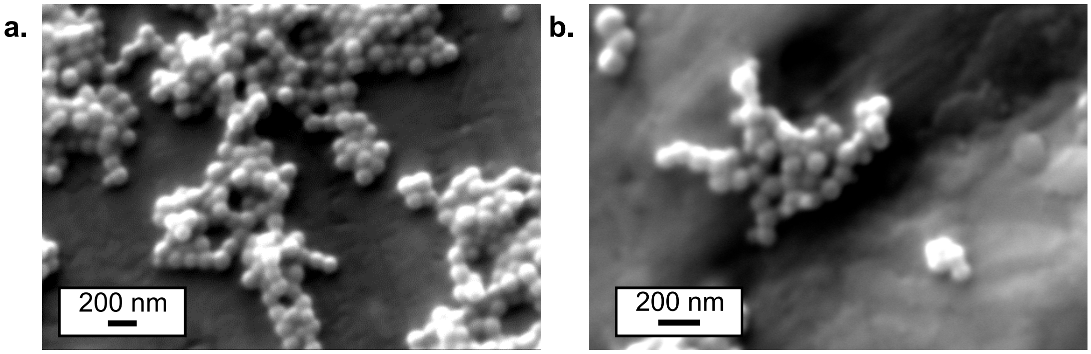

Figure S1. Scanning electron microscopy images confirm the manufacturer reported diameters and spherical morphology of the SO_4-PSNPs (a) and COOH-PSNPs (b) used in this study.

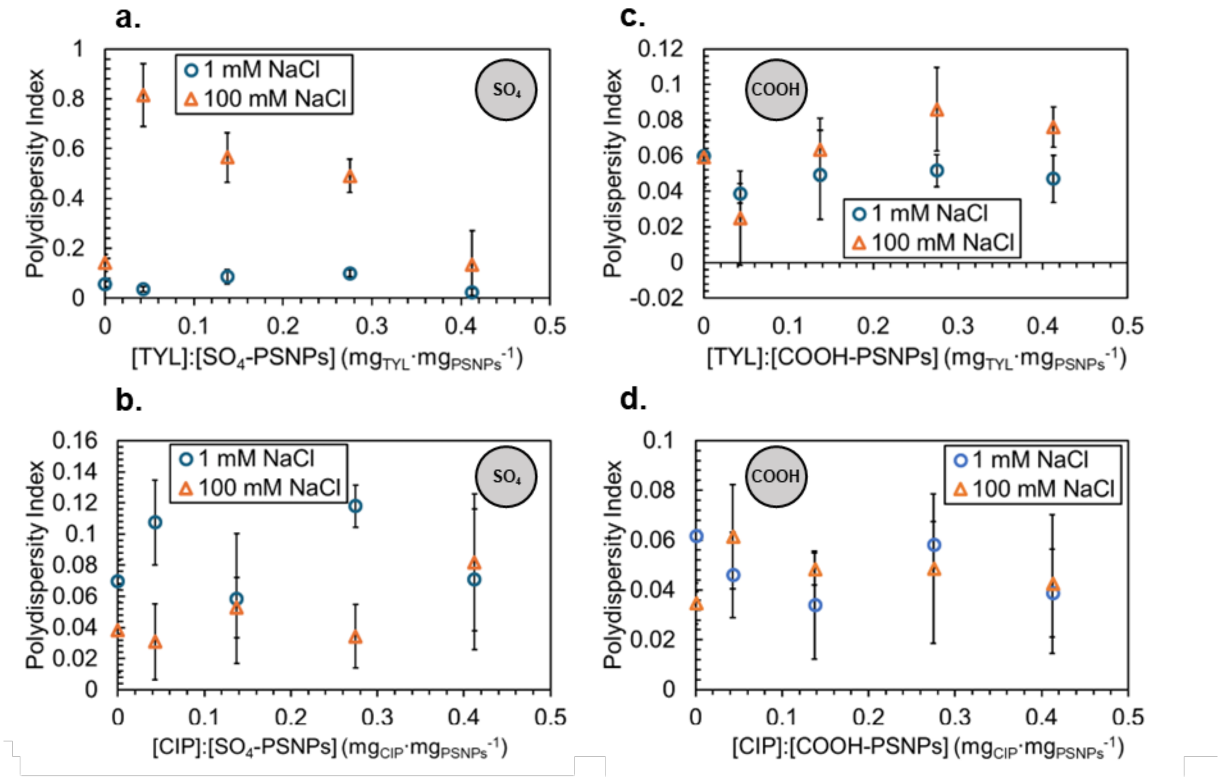

Figure S2. Polydispersity index for SO_4-PSNPs (a,b) and COOH-PSNPs (c,d) as a function of [TYL]:[PSNP] ratio (a,c) or [CIP]:[PSNP] (b,d).

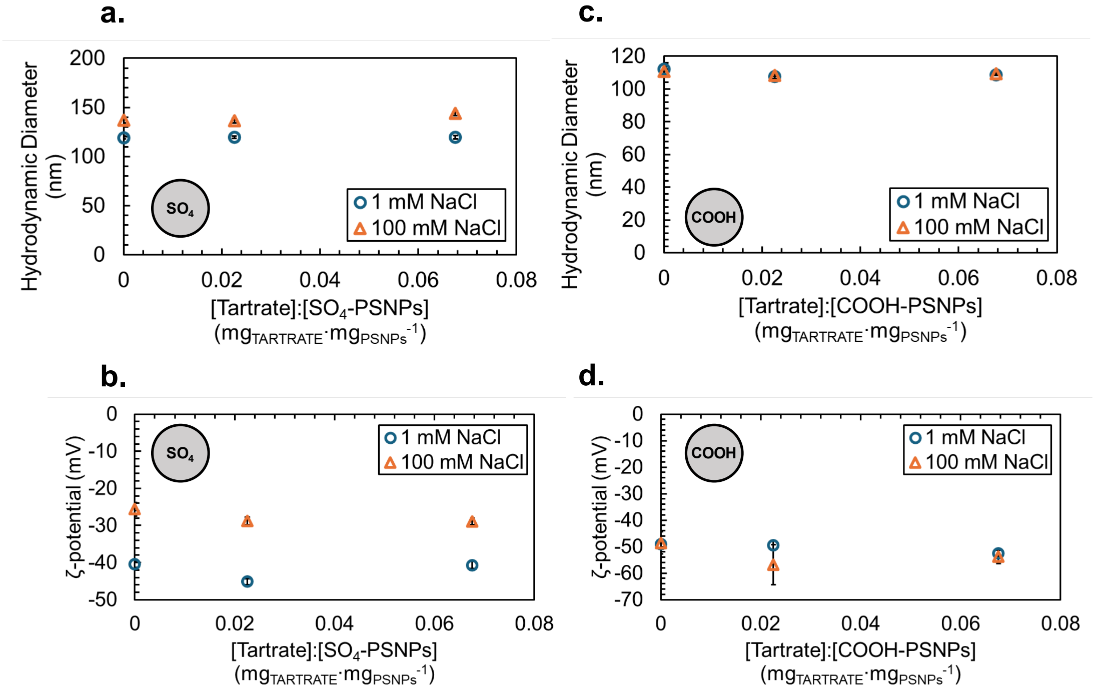

Figure S3. Hydrodynamic (a,c) and electrokinetic (b,d) characterization of SO_4-PSNPs (a,b) and COOH-PSNPs (c,d) as a function of [tartrate]:[PSNP] ratio to show that any changes observed to the particles in the presence of tylosin tartrate are by the tylosin in solution not the tartrate counterion.

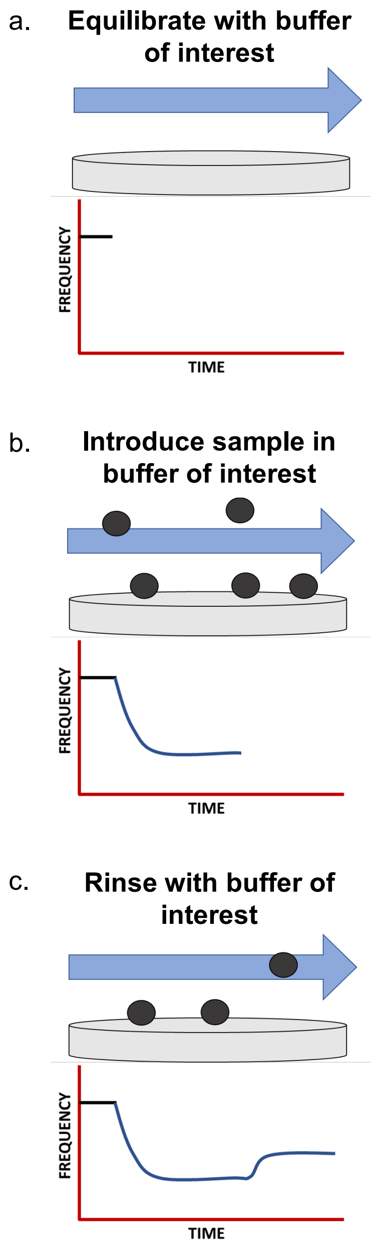

Figure S4. Schematic showing the work flow of the QCM-D experiments to measure mass deposited of either COOH-PSNPs or SO_4-PSNPs in the presence and absence of tylosin or ciprofloxacin. a. The system is equilibrated in buffer of interest until a stable baseline is reached. b. The desired sample (PSNPs, antibiotic, or PSNPs + antibiotic) is flowed over the SiO_2 sensor until a stable frequency is reached. c. The surface is rinsed with buffer that does not contain the analyte(s) of interest to rinse away any loosely bound mass.

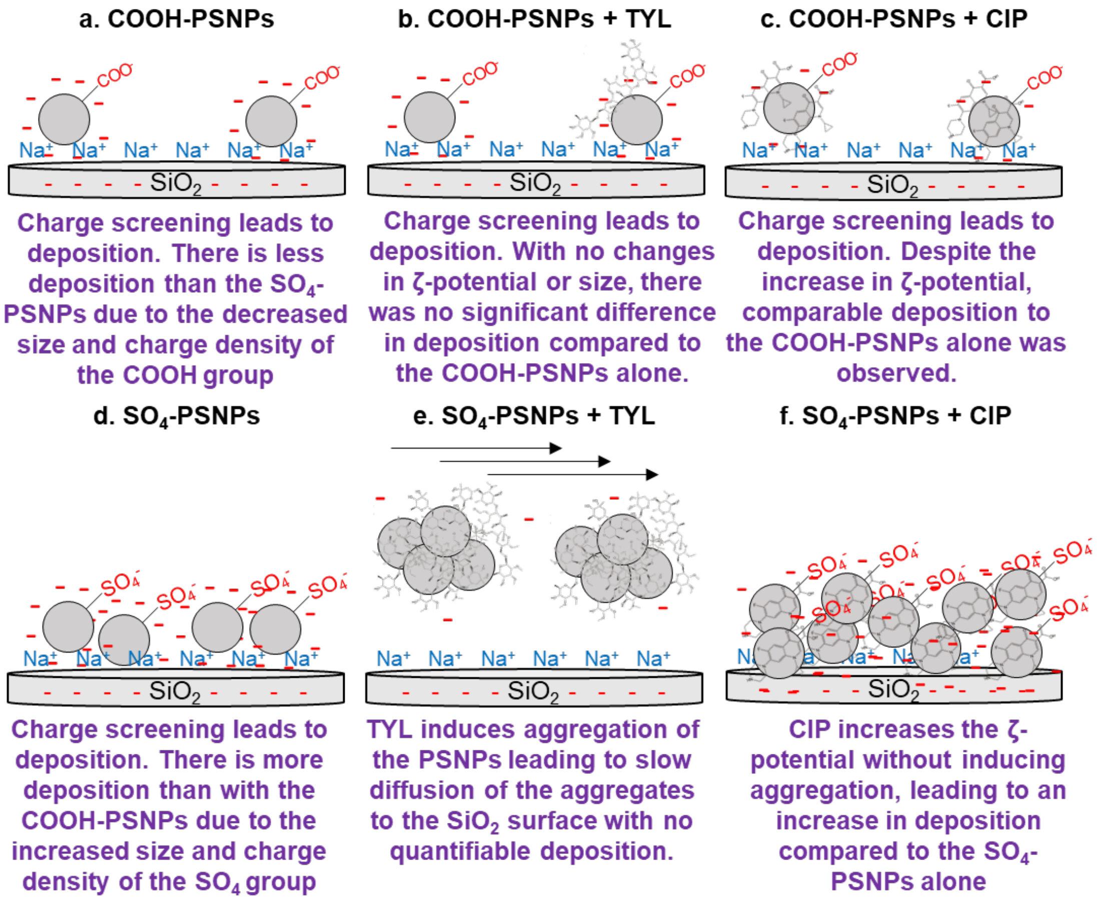

Figure S5. Schematic showing hypothesized models for deposition for a. COOH-PSNPs, b. COOH-PSNPs + TYL, c. COOH-PSNPs + CIP, d. SO_4-PSNPs, e. SO_4-PSNPs + TYL, f. SO_4-PSNPs + CIP. In all scenarios the solution conditions were 10 mM HEPES, 100 mM NaCl, pH 7.4.

Table S1. Summary of mass deposited ($ng{\cdot}cm^2$) prior to and after rinse with buffer for each of the pollutant combinations noted at either 1 mM NaCl or 100 mM NaCl. In both cases deposition was monitored with 10 mM HEPES at a pH of 7.4. ND refers to values below the detection limit of the QCM-D (~5 $ng{\cdot}cm^2$)

Buffer Conditions	Pollutant(s) Added	Maximum mass deposited prior to rinse ($ng{\cdot}cm^2$)	Mass deposited post rinse ($ng{\cdot}cm^2$)
1 mM NaCl	COOH-PSNPs only	ND	ND
	COOH-PSNPs + TYL	ND	ND
	COOH-PSNPs + CIP	25 ± 2	ND
	SO_4-PSNPs only	ND	ND
	SO_4-PSNPs + TYL	ND	ND
	SO_4-PSNPs + CIP	30 ± 4	7 ± 6
	TYL only	ND	ND
	CIP only	29 ± 17	ND
100 mM NaCl	COOH-PSNPs only	66 ± 3	31 ± 13
	COOH-PSNPs + TYL	30 ± 20	26 ± 18
	COOH-PSNPs + CIP	180 ± 40	60 ± 40
	SO_4-PSNPs only	190 ± 80	170 ± 40
	SO_4-PSNPs + TYL	ND	ND
	SO_4-PSNPs + CIP	400 ± 140	290 ± 180
	TYL only	ND	ND
	CIP only	ND	ND
